# René Krebs: Prüfen mit Multiple Choice: Kompetent planen, entwickeln, durchführen und auswerten

**DOI:** 10.3205/zma001274

**Published:** 2018-02-15

**Authors:** Lambert Schuwirth

**Affiliations:** 1 Flinders University, Prideaux Research Centre, Adelaide, Australia

## Bibliographical details

René Krebs

**Prüfen mit Multiple Choice: Kompetent planen, entwickeln, durchführen und auswerten**

Publisher: Hogrefe

Year of publication: 2019, pages: 184, prize: 34,95 €

## Recension

The book “Prüfen mit Multiple Choice” is clearly a must for anyone who is involved in assessment in the health professions, but clearly also far beyond this domain; it is a rich source of complete information for anyone who is involved in multiple-choice assessment. I have never seen a book that is so incredibly clear and accessible and at the same time so complete. But these are not the only qualities it combines: it is both practical and evidence informed. This book can both be used as a how-to guide and as an entry into the underlying evidentiary basis. I could not think of anyone involved in assessment who would NOT benefit from possessing this book.

The writing is clear; the author has refrained from using overly long sentences. This makes it easy to read. The book is subdivided into four sections: background of multiple choice testing, productions of multiple-choice questions, different types of multiple choice questions and quality assurance and improvement processes. Each section is subdivided into meaningful subsections and the pull outs of keywords in the margin allows for quick navigation.

**Figure 1:**
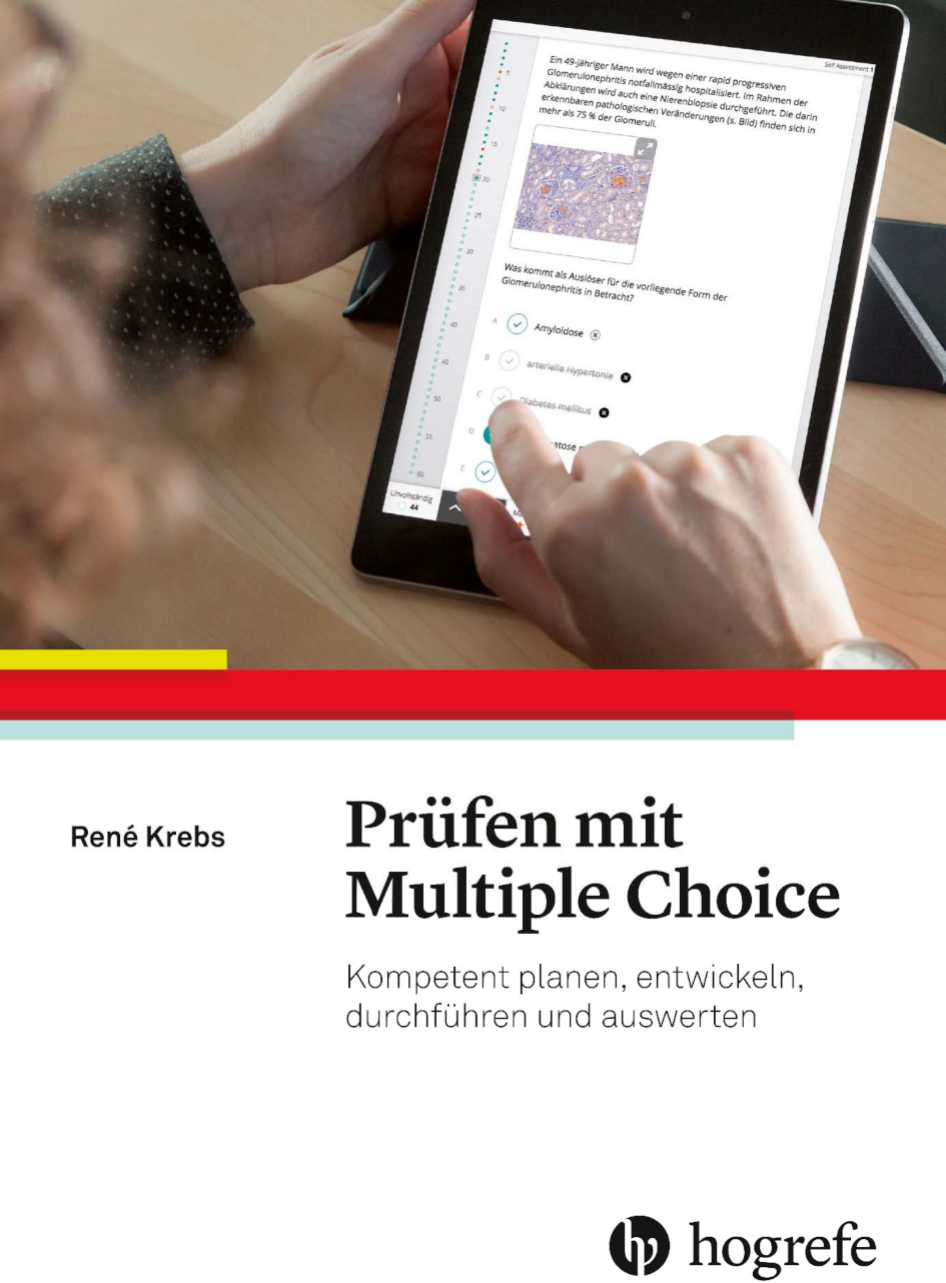
Bookcover

I am glad to have received a first copy of it and I am sure I will be using it in my work and recommending it to others (see figure 1 (Fig. 1)).

## Competing interests

The author declares that he has no competing interests.

